# A stone miner with both silicosis and constrictive pericarditis: case report and review of the literature

**DOI:** 10.1186/1471-2466-13-71

**Published:** 2013-12-06

**Authors:** Yajian Jiang, Fangchun Shao

**Affiliations:** 1 Department of Pulmonology, Sir Run Run Shaw Hospital, Zhejiang University School of Medicine, 3 East Qingchun Road, Hangzhou, Zhejiang 310016, China; 2 Program in Clinical Medicine, School of Medicine, Zhejiang University, Hangzhou, Zhejiang, China

**Keywords:** Stone miner, Silicosis, Constrictive pericarditis, Immune response

## Abstract

**Background:**

The working environment of stone miners has been believed to cause their susceptibility to respiratory diseases. Silicosis is an occupational disease caused by exposure to crystalline silica dust which is marked by inflammation and scarring in the lung. The immune system boosted after the silica invasion led to self-damage and lay the foundation of silicosis pathogenesis. Silicosis coexisting with other diseases in one patient has been reported, however, was not reported to coexist with constrictive pericarditis. We, for the first time, reported a patient with silicosis and constrictive pericarditis and thought the immune response was probably the link between the two.

**Case presentation:**

A 59-year-old Chinese stone miner complained of chest distress was found to have lung nodules which were found to be silica deposits by biopsy. This patient was also found to have constrictive pericarditis at the same time. Later surgical decortication cured his symptoms.

**Conclusion:**

We provided the first case having constrictive pericarditis concomitant with silicosis. A probable link between the two diseases was the immune response boosted by the silica deposits.

## Background

Stone mining is a profession with high risks to occupational lung disease due to its harmful working environment [1,2]. Among all the notorious pathogens present at stone miner’s working site, silica dust is believed to be the responsible agent causing the disease silicosis, a worldwide occupational lung disease. Exposure to crystalline silica dust leads to inflammation and scarring of the lung tissue and ultimately respiratory insufficiency [3]. Pathogenesis of silica deposits to the lung has been widely studied, however, with no conclusive mechanism being reached. Some believed the silica deposits could cause immune response which was responsible for silicosis progression [4,5] while some others believed the trace metals found on silica dusts played the major role in silicosis pathogenesis [6,7]. Later the detection of micro-organisms attached to the silica dust suggested another possibility that the onset and progression of silicosis might be determined by the micro-organisms [8-10]. Moreover, silicosis is found to be not only a respiratory disease, but also can be associated with other disorders. It has been reported that silicosis was associated with lupus-like autoimmune disease [11] and silicosis could complicate existing diseases such as lung cancer [12]. Others found pericardial plague as a complication of silicosis in one patient [13]. Herein, we report a patient with silicosis associated with constrictive pericarditis. A detailed case will be presented and the association between the two diseases will be discussed below.

## Case presentation

A 59-year-old male who used to work as a stone miner for 10 years complained of chest distress and dyspnea for months and worsened 3 days before admission. Symptoms were aggravated either after activities or lying flat. He had no chest pain, hemoptysis, fevers, chills, night sweats and his vital signs were stable before admission. Patient reported a smoking history of 30 years. Rales or wheezes were not heard during auscultation.

**Figure 1:**
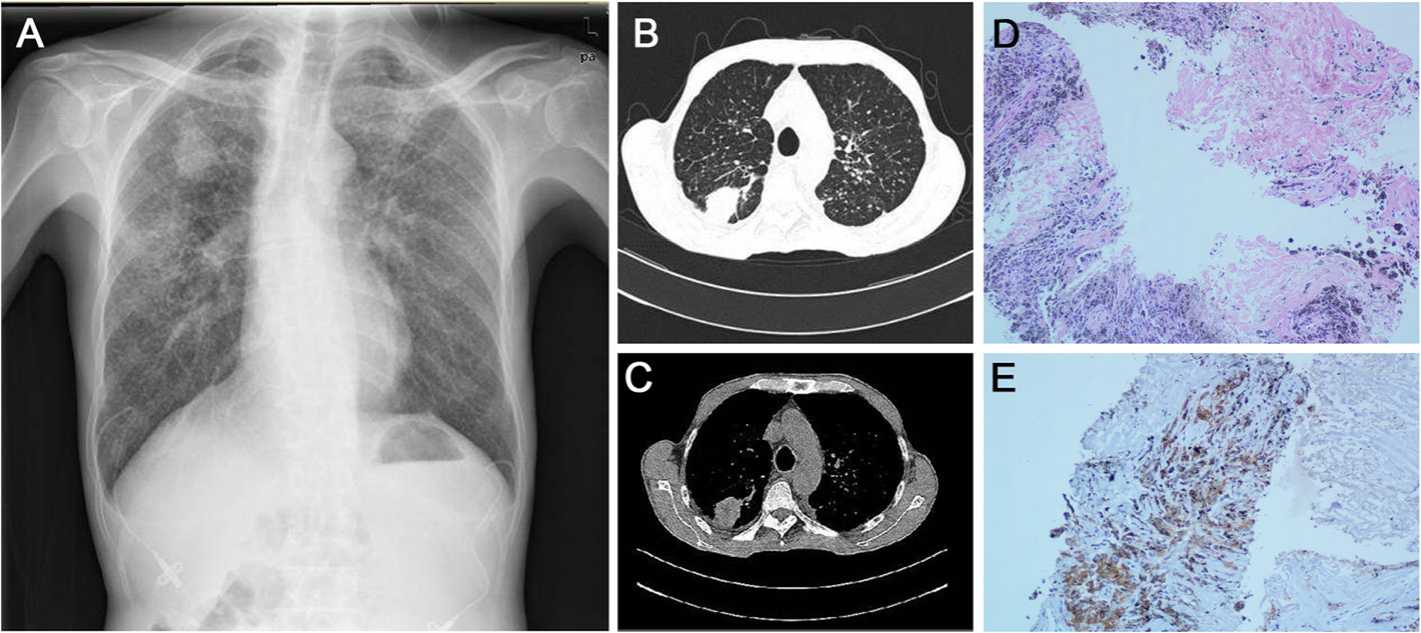
**Imaging and histology of the nodule.** Chest X-ray **(A)** and CT scan **(B&C)** showed a 25×33 mm nodule in the right upper lung. Diffused small nodules throughout the lung can be seen in the chest-X ray. HE (hematoxylin and eosin) staining **(D)** of the biopsied 25×33 nodule showed fibrous tissue hyperplasia and hyaline change. Carbon deposits and chronic inflammatory cell infiltration could be seen. Immunostaining **(E)** of CD163 and CK (criatinne kinase) showed positive CD163 staining and negative CK staining.

**Figure 2:**
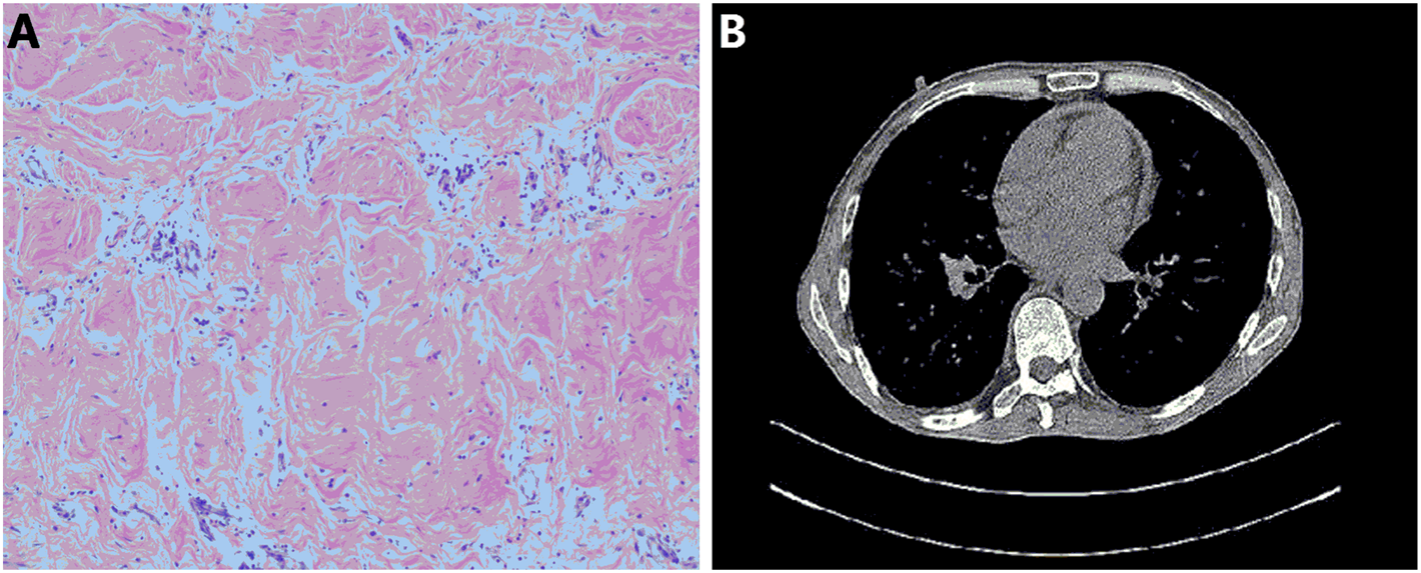
**Imaging and histology of the restrictive pericardium.** HE staining **(A)** of excised pericardium demonstrated granular and fibrous tissue hyperplasia. Acid fast stain was negative. Chest CT **(B)** found a thickened and constrictive pericardium.

Initial investigations including chest X-ray and CT found a 25×33 mm nodule in the right upper lung and diffused small nodules throughout the lung (Figure 1). The chest CT also demonstrated thickened and restricted pericardium (Figure 2). CT guided biopsy of the 25×33 cm lung nodule was performed and pathology demonstrated fibrous tissue hyperplasia and hyaline changes with carbon deposits and chronic inflammatory cell infiltration (Figure 1), which were definite pathologic evidences of silicosis. No tuberculosis bacilli were found in the tissues. We then had this patient screened for TB (tuberculosis) antibodies, T-spot.*TB* and PPD skin test. However, none of these TB screens were positive.

The patient, diagnosed with silicosis and constrictive pericarditis, was then transferred to surgery department for pericardium decortication. The surgery went successfully. Pathology of the excised pericardium showed granular and fibrous tissue hyperplasia and negative fast acid staining (Figure 2). Patient was well recovered from previous dyspnea and chest distress and discharged afterwards. He remained very well and reported no similar symptoms during our follow-ups. Follow-up chest X-rays did not show any enlargement of the 25×33 mm nodule in the right upper lung.

## Discussion

Silicosis remains to be a worldwide occupational lung disease despite global efforts of prevention such as reducing silica dust concentration in the working environment and wearing protective masks [1,2]. The disease is caused by inhalation of silica dust into the lung. When fine particles of silica dust are deposited in the lung, macrophages will ingest the dust particles and start an inflammatory response by releasing molecules like tumor necrosis factors, interleukin1, leukotriene B4 and other cytokines. These released molecules stimulate fibroblasts to proliferate and produce collagen around the silica particle, thus resulting in fibrosis and the formation of nodular lesions [4,5,14]. After depositing into the lung, the silica particles will form fibrotic nodules with concentric arrangement collagen fibers induced by self-immune elimination mechanism, thus leading to microscopic central hyalinization and a cellar peripheral zone [1,3].

It is not done simply by depositing the silica particles in the lung. The surface of silica dust can generate radicals which can damage the surrounding cells [15]. Moreover, trace metals attached to the silica dusts may also influence the damage to lung tissue of the silica dusts and determine the onset and progression of silicosis. Previous reports showed that the gold or foundry miners needed less silica to get silicosis than those who exposed to pure silica [6] and the importance of trace metals in silicosis pathogenesis was later confirmed by studies in Chinese tin and tungsten workers [16]. In addition, micro-organisms such as tuberculosis bacilli attached to the silica dusts may also contribute to the pathogenesis and progression of silicosis, which was reported earlier [8-10].

In this report, our patient, with a typical occupational history of stone mining for a decade and suspicious chest imaging results, was diagnosed silicosis by definite pathology results. Surprisingly, our patient was found to have coexisting constrictive pericarditis based on both chest CT and echocardiography. Could there be some underlying associations between the two diseases or they are just two independent diseases coincidentally exist in this patient? The reasons for constrictive pericarditis can be idiopathic or viral (42%-49%), post-infectious (tuberculous or purulent pericarditis, 3%-6%), post-cardiac surgery (11%-37%), post-radiation therapy (9%-31%), connective tissue disorders (3%-7%), and unknown etiologies [17,18]. For our patient in this case, the most probable cause would be post-infectious since silicosis is a potent state for all kinds of lung infection such as tuberculosis, though other causes would still be possible. However, we failed to find evidences supporting the post-infection hypothesis through a negative tuberculosis screening and a negative chest imaging for infectious.

Although in our patient, the underlying association between silicosis and constrictive pericarditis remains obscure, there have been several articles reporting cases of silicosis associating with other diseases. RA Wilke et al. reported a case with lupus-like autoimmune disease associated with silicosis [11]. The authors suggested that the association could be the result of an immune response aroused by granulomatous silicotic nodular components. The immune response ultimately caused autoimmune disease, as in this patient the lupus-like disease affecting the skin and kidney. I Mohebbi et al. reported a pericardial plague seen in a patient with silicosis [13]. Pathology of the pericardial specimen showed typical basket-weave collagen pattern suggesting silica deposits. However, the authors were not sure how silica was released to the pericardium and induced the later damage.

For our patient having constrictive pericarditis and silicosis, there may be potential underlying associations between the two. Based on the etiology of constrictive pericarditis, we screened the patient for tuberculosis infection, however the results came negative. As a growing number of evidences supporting extra-pulmonary symptoms in silicosis due to immune reactions and extra-pulmonary deposits of silica, it is still highly possible that for our patient, the constrictive pericarditis is associated with silicosis. One possible explanation would be that the silica deposits broke the immune homeostasis and induced heavy immune reaction not only restricted to the lung, but also in the pericardium. This immune reaction ultimately caused the pericardial damage. Another possible explanation would be the release of silica to the pericardium in a somehow unknown way, thus causing damage to the pericardial tissue. A third explanation would be our silicotic patient had some kind of infection that led to constrictive pericarditis, and then was cured of the infection leaving no detectable clues for us. With our current understanding, it requires investigation in depth of silicosis and its associating pathogenesis. The exact association of silicosis and pericardial disease remains unknown, pericardial damage can be possibly immune reaction induced by inhaled silica, or translocation of silica dusts through pulmonary capillaries, or post-silicosis infection.

Yet our patient was diagnosed with silicosis for certain, lung cancer still remained on the possible disease list of this patient. Especially when this patient had many years’ smoking history and the big nodules present on his chest CT, lung cancer would still be possible despite a one-time biopsy. However, at that point, negative bronchoscopy showing no obstruction nor cancer cells in the brushed specimen indicated the main cause of patient distress symptoms was his pericardium constriction. A better way to rule out cancer possibility was a strict follow-up after surgery. After surgery, patient got released from previous symptoms and did a monthly follow-up with our team. Chest CT was scheduled half a year. No signs of enlargement of the previous biggest nodule or increasing nodules were found by chest imaging further lowering the cancer possibility.

## Conclusion

We report a male stone miner patient having both silicosis and constrictive pericarditis. Screening of tuberculosis and other possible infections is negative. Patient remained well after surgical pericardial decortication and was still in our follow-up for his silicosis condition.

## Consent

Written informed consent was obtained from the patient for publication of this Case report and any accompanying images. A copy of the written consent is available for review by the Editor-in-Chief of this journal.

## Abbreviations

TB: Tuberculosis; HE: Hematoxylin and eosin; CK: Criatinne kinase.

## Competing interests

There are no conflicts of interest between the authors, the authors and the patient.

## Authors’ contributions

YJ drafted this manuscript with FS supervision. YJ took care of this patient in a medical team led by FS. Both authors read and approved the final manuscript.

## Pre-publication history

The pre-publication history for this paper can be accessed here:

http://www.biomedcentral.com/1471-2466/13/71/prepub
